# Public perception on the role of community pharmacists in self-medication and self-care in Hong Kong

**DOI:** 10.1186/1472-6904-11-19

**Published:** 2011-11-25

**Authors:** Joyce H You, Fiona Y Wong, Frank W Chan, Eliza L Wong, Eng-kiong Yeoh

**Affiliations:** 1 School of Pharmacy, The Chinese University of Hong Kong, Shatin, N.T., Hong Kong; 2 School of Public Health and Primary Care, The Chinese University of Hong Kong, Prince of Wales Hospital, Shatin, N.T., Hong Kong

## Abstract

**Background:**

The choices for self-medication in Hong Kong are much diversified, including western and Chinese medicines and food supplements. This study was to examine Hong Kong public knowledge, attitudes and behaviours regarding self-medication, self-care and the role of pharmacists in self-care.

**Methods:**

A cross-sectional phone survey was conducted, inviting people aged 18 or older to complete a 37-item questionnaire that was developed based on the Thematic Household surveys in Hong Kong, findings of the health prorfessional focus group discussions on pharmacist-led patient self management and literature. Telephone numbers were randomly selected from residential phone directories. Trained interviewers invited eligible persons to participate using the "last birthday method". Associations of demographic characteristics with knowledge, attitudes and beliefs on self-medication, self-care and role of pharmacists, and spending on over-the-counter (OTC) products were analysed statistically.

**Results:**

A total of 1, 560 phone calls were successfully made and 1, 104 respondents completed the survey which indicated a response rate of 70.8%. 63.1% had adequate knowledge on using OTC products. Those who had no formal education/had attended primary education (OR = 3.19, 95%CI 1.78-5.72; p < 0.001), had attended secondary education (OR = 1.50, 95%CI 1.03-2.19; p = 0.035), and aged ≥60 years (OR = 1.82, 95% CI 1.02-3.26; p = 0.042) were more likely to have inadequate knowledge on self-medication. People with chronic disease also tended to spend more than HKD100 on western (OR = 3.58, 95%CI 1.58-8.09; p = 0.002) and Chinese OTC products (OR = 2.94, 95%CI 1.08-7.95; p = 0.034). 94.6% believed that patients with chronic illnesses should self-manage their diseases. 68% agreed that they would consult a pharmacist before using OTC product but only 45% agreed that pharmacists could play a leading role in self-care. Most common reasons against pharmacist consultation on self-medication and self-care were uncertainty over the role of pharmacists and low acceptance level of pharmacists.

**Conclusions:**

The majority of respondents supported patients with chronic illness to self-manage their diseases but less than half agreed to use a pharmacist-led approach in self-care. The government should consider developing doctors-pharmacists partnership programs in the community, enhancing the role of pharmacists in primary care and providing education to patients to improve their awareness on the role of pharmacists in self-medication and self-care.

## Background

Self-care is defined by World Health Organization (WHO) as activities that individuals, families and communities undertake with the intention of enhancing health, preventing disease, limiting illness, and restoring health [1]. Self-care and self-medication have attracted considerable international healthcare policy interest, because they do not only effectively reduce the burden on health services, but also improve compliance and disease outcome [2,3]. These have significant implications to the health system and society at large, as poor adherence to drug regimes among patients would lead to considerable economic and social costs [4]. Evidence suggests that self-care skill-orientated programmes may be more effective than information-only patient education in improving clinical outcomes and reducing health care costs [2]. In the United Kingdom, it had been a new approach to Chronic Disease Management for the 21^st ^Century. The Expert Patient Programme, which aims to introduce lay led self-care training for patients, is expected to become one of a range of integrated self-care options in health and social care for people with long-term conditions [5].

Medication compliance is one of the important elements in self-care. It is common for patients to use over-the-counter (OTC) medicines without the supervision of healthcare professionals, which can limit the opportunity for ongoing patient follow-up and safety monitoring. The establishment of a robust pharmacovigilance system is therefore advocated, in which pharmacists play an important role in providing advice to patients when they purchase OTC drugs [6]. In the UK, there is also a move to promote the role of pharmacists and develop a broader concept of the primary care team [7-9]. Pharmacist's role has been extended to tobacco cessation therapy, local health promotion, advice to family doctors and other health professionals, repeated prescription, advice to nursing and residential homes, health screening and diagnosis, etc [10]. Meanwhile, general practitioners have also become more supportive of pharmacists' extended role in western countries [11,12].

Of the seven-million population in Hong Kong, approximately 20.2% reported to have diseases that required long-term follow-up [13]. The care of patients with chronic illness is a substantial burden to the Hong Kong health system as well as to their care-givers [14]. The choices for self-medication in Hong Kong are much diversified, including western medicines, Chinese medicines and food supplements. The wide spectrum of OTC products potentiates adverse drug events and undesirable drug interactions, particularly in patients receiving chronic drug therapy. The role of pharmacists in Hong Kong, however, mainly focuses on medication management and is much more limited compared to many western countries. To better understand the public perspective on these issues and to develop a policy framework on pharmacist-led self-management in the future, we therefore examined public knowledge, attitudes and behaviour regarding self-medication and self-care of chronic illness as well as the role of community pharmacists in patient self-care and self-medication in Hong Kong.

## Methods

### Study design and setting

This study was based a cross-sectional phone survey on a random sample of approximately 1, 100 non-institutionalized Hong Kong residents. The survey was conducted by trained interviewers from the telephone survey service team based at the School of Public Health and Primary Care, CUHK. The interviewers had experiences in conducting numbers of telephone surveys and they were supervised by a project coordinator to ensure survey quality. All phone calls were made between 6:30 pm and 10 pm, during the period of June to July, 2009. Hong Kong permanent residents aged ≥18 years who were able to communicate in Cantonese were eligible to participate in the survey. Telephone numbers were randomly selected from residential phone directories. Interviewers, after briefing the purpose of the study, invited eligible persons to participate in the survey using "the last birthday method". Household member at least 18 years of age, whose birthday was closest to the date of the interview, was invited to complete the survey. Verbal consent was obtained in advance.

The interview was conducted in Cantonese for about 10-15 minutes. Those who were in the city for vacation, or were incapable of responding to survey questions because of psychiatric or neurological disorders were excluded. The interview was pre-tested with 10 subjects. Appropriate adjustments were made before it was administered in the main study.

### Instruments

Since a validated pre-developed questionnaire could not be identified, the questionnaire was developed based on the Thematic Household Surveys in Hong Kong [15,16], health professional focus group discussions and literature. It consisted of a total of 37 questions divided into 5 sections: (1) knowledge and attitude on self-medication, (2) utilization and expenses on OTC products including western medicine, Chinese medicine and food supplement, (3) behaviour and attitude on self-care of chronic illnesses, (4) role of pharmacist in patient self-care of chronic illnesses and self-medication, and (5) respondent's demographics. Questions on knowledge and attitude were responded with three choices: agree, no comment/don't know, and disagree. Open-ended questions to explore the reasons of agreeing, no comment and disagreeing of self-care of chronic illnesses, and role of pharmacist in self-care and self-medication, were also developed. The reasons given were further categorized in the analysis. There were a total of six questions on knowledge. "One" point was given for answering each question correctly which gave a full score of 6. OTC products were described as western and Chinese medications and supplements which could be purchased without physicians' prescription. The tasks of pharmacists in pharmacist-led patient self-care of chronic diseases were described as handling of drug-related issues, monitoring the effectiveness and safety of drug treatment, providing information/education on drug therapy and life-style modification, and performing medical triage services. Chronic diseases were explained to the respondents as long-term diseases diagnosed by physicians. They were asked to name the chronic disease(s) they were diagnosed of. A total of 19 common chronic diseases were listed on the questionnaire. If the disease mentioned was not on the list, the interviewers would mark it down and investigators of this project, who were clinicians, would judge whether the disease was a chronic one.

The questionnaire had been commented and revised by experts and pilot-tested before implementation in the main study.

The study protocol was approved by the Joint CUHK-NTEC Clinical Research Ethics Committee and was performed in accordance with the World Medical Association's Declaration of Helsinki.

### Statistical analysis

Descriptive statistics were reported by mean ± standard deviation or percentage, as appropriate. The Hong Kong population size was 6.8 million in mid-2006 [17]. Assuming a prevalence rate of 50%, a sample size of 1100 would provide a precision of 3% from the true values at 95% confidence level. Associations of socio-demographic variables with knowledge, attitudes/beliefs on self-medication, self-care and role of pharmacists, and spending on OTC products were first evaluated using univariate analysis by logistic regression. Factors with significant association in the univariate analysis were further analyzed by stepwise multiple logistic regression analysis. A p-value of < 0.05 was considered as statistically significant.

## Results

### Characteristics of the study population

A total of 1, 560 phone calls were successfully made and 1, 104 (70.8%) respondents met the selection criteria and completed the survey. Socio-demographic data of the respondents were shown in Table 1. There were 532 (48.2%) male respondents and the largest age group was 30 to 49 years (39.7%).

**Table 1:** Demographic characteristics of 1, 104 respondents

	Number
Age (years) (N = 1, 103)*	
18-29	218
30-49	438
50-69	334
≥70	113
**Male**	532
**Presence of chronic disease(s)**	215
Education (N = 1, 096)*	
Primary or below	164
Secondary	604
Tertiary or above	328
Family monthly income (HKD) (N = 823)^# ^*	
≤ $5, 999	83 (10.1)
$6, 000- $9, 999	73 (8.9)
$10, 000-$29, 999	424 (51.5)
$30, 000-$59, 999	172 (20.9)
$60, 000 or above	71 (8.6)

*Total number < 1, 104 because of missing data; ^#^HKD1 = USD0.128

### Knowledge and attitude on self-medication

A majority of respondents had correct knowledge on the need to seek medical care when symptoms continues despite the use of OTC products (98.5%), awareness of drug-food interactions (86.8%) and not to share medications with others who have similar symptoms (84.5%). Over half of the respondents agreed that the OTC products may mask the symptoms of severe underlying diseases (65.2%). Only 23.1% of respondents were correct about Chinese OTC products not necessarily cause less adverse effects than western OTC products. Few respondents (12.6%) agreed that western OTC products can be concurrently used with Chinese OTC products. A total of 1093 respondents completed all the six knowledge questions and 690 (63.1%) scored 4 or above. A cut-off point of 4 was chosen as the mean of knowledge score was 3.71 ± 0.99. Multiple logistic regression analysis showed that lower education level, including no schooling/primary education (OR = 3.19, 95%CI 1.78-5.72; p < 0.001) and secondary education (OR = 1.50, 95%CI 1.03-2.19; p = 0.035), and patients aged 60 years or above (OR = 1.82, 95% CI 1.02-3.26; p = 0.042) were associated with higher odds of inadequate knowledge score (less than 4) (Table 2).

**Table 2:** Factors affecting knowledge score on self-medication

	Univariate analysis (Chi-square) Knowledge Score				Multivariate analysis (Logistic regression) Knowledge Score < 4	
	**< 4**	**≥4**	**X^2^**	**P**	**OR (95% CI)**	**P**
	**n (%)**	**n (%)**				

**Age**						
18-29	64 (15.9)	154 (22.4)	49.05	< 0.001	reference	0.893
30-59	208 (51.6)	434 (63.0)			0.97 (0.64-1.47)	0.042
60 or above	131 (32.5)	101 (14.7)			1.82 (1.02-3.26)	
**Gender**						
Male	191 (47.5)	340 (49.3)	0.316	0.616	0.91 (0.67-1.23)	0.529
Female	211 (52.5)	350 (50.7)			reference	
**Education**						
No schooling/primary	100 (25.1)	61 (8.9)	64.74	< 0.001	3.19 (1.78-5.72)	< 0.001
Secondary	218 (54.6)	379 (55.2)			1.50 (1.03-2.19)	0.035
Diploma/degree	81 (20.3)	246 (35.9)			reference	
**Monthly household income (HK$)**^#^						
< $2000-$5999	37 (13.4)	45 (8.3)	11.98	0.007	0.90 (0.48-1.68)	0.746
$6000-$9999	27 (9.8)	46 (8.5)			0.99 (0.55-1.78)	0.973
$10000-$24999	130 (47.1)	231 (42.5)			1.20 (0.83-1.73)	0.336
≥$25000	82 (29.7)	221 (40.7)			reference	
**Presence of chronic illness**						
Yes	309 (76.9)	571 (82.9)	5.88	0.017	1.03 (0.68-1.57)	0.877
No	93 (23.1)	118 (17.1)			reference	

^#^HKD1 = USD0.128

### Utilization and expenses on OTC products

Over the past three months, 363 of 1102 (32.9%) respondents had purchased OTC products. Among these 363 OTC product users, the numbers of people who used western OTC medications only, Chinese OTC products only and food supplement only including vitamins and minerals were 150 (41.3%), 43(11.8%) and 118 (32.5%), respectively. Besides, the numbers of people who used western and Chinese OTC products, western OTC products and food supplement, Chinese OTC products and food supplement, and western and Chinese OTC products and food supplement were 24 (6.6%), 16 (4.4%), 6 (1.7%) and 6 (1.7%), respectively. The most commonly used western OTC products were common cold medicines (108/196; 55.1%) and oral analgesics (73/196; 37.2%). The most commonly used Chinese OTC products were proprietary medicines (27/79; 34.2%) and herbal medicines (25/79; 31.56%). Most of the respondents spent HKD 100 or less in purchasing western OTC products (80.7%) and Chinese OTC products (48%), while 59.2% spent HKD 101-500 in food supplement including vitamins and minerals (HKD1 = USD0.128). As the proportion of spending HKD100 versus over HKD100 was approximately 50/50, therefore, the breakpoint of HKD100 was selected for the analysis. Multiple logistic regression analysis showed that the presence of chronic disease was a significant predictor of spending over HKD100 on western OTC products (OR = 3.58, 95%CI 1.58-8.09; p = 0.002) and Chinese OTC products (OR = 2.94, 95%CI 1.08-7.95; p = 0.034) (Table 3). No influential factor was identified on the spending of food supplement.

**Table 3:** Factors affecting amount spent in purchasing western and Chinese OTC products

	Western OTC products						Chinese OTC products					
		**Univariate analysis (Chi-square)**			**Multivariate Analysis (Logistic Regression)**		**Univariate analysis (Chi-square)**			**Multivariate Analysis (Logistic Regression)**		

	**≤ $100** **n (%)**	**$101 or above** **n (%)**	**X^2^**	**P**	**$101 or above** **OR (95% CI)**	**P**	**≤ $100** **n (%)**	**$101 or above** **n (%)**	**X^2^**	**P**	**$101 or above** **OR (95% CI)**	**P**

**Age (yrs)**												
18-29	34 (22.5)	5 (13.9)	1.61	0.446			5 (14.3)	4 (10.3)	0.28	0.868		
30-59	99 (65.6)	25 (69.4)					23 (65.7)	27 (69.2)				
60 or above	18 (11.9)	6 (16.7)					7 (20.0)	8 (20.5)				
**Gender**												
Male	74 (49.3)	12 (33.3)	2.99	0.096	0.54 (0.25-1.19)	0.126	18 (50.0)	12 (30.8)	0.29	0.104	0.50 (0.19-1.31)	0.157
Female	76 (50.7)	24 (66.7)			reference		18 (50.0)	27 (69.2)			reference	
**Education**												
No schooling/primary	14 (9.3)	3 (8.3)	0.11	0.949			5 (14.3)	8 (21.1)	0.86	0.651		
Secondary	79 (52.7)	20 (55.6)					18 (51.4)	20 (52.6)				
Diploma/degree	57 (38.0)	13 (36.1)					12 (34.3)	10 (26.3)				
**Monthly household income (HK$)**^#^												
< $2000-$5999	7 (5.4)	2 (8.0)	3.05	0.384			1 (3.8)	4 (14.8)	2.16	0.339		
$6000-$9999	12 (9.2)	0					0	0				
$10000-$24999	56 (43.1)	10 (40.0)					15 (57.7)	12 (44.4)				
≥$25000	55 (42.3)	13 (52.0)					10 (38.5)	11 (40.7)				
**Presence of chronic illness**												
Yes	22(14.6)	14 (38.9)	11.06	0.002	3.58 (1.58-8.09)	0.002	9 (25.0)	20 (51.3)	5.45	0.032	2.94 (1.08-7.95)	0.034
No	129 (85.4)	22 (61.1)			reference		27 (75.0)	19 (48.7)			reference	

^#^HKD1 = USD0.128

Questions on attitudes/beliefs of self-medication using OTC products indicated that 30.8% (339/1100) respondents believed that OTC products should be used at the occurrence of first sign/symptom. Less than half of the respondents believed that OTC products were effective (390/1098; 35.5%) or safe (490/1099; 44.6%). Most of respondents (983/1093; 89.8%) would follow instructions on package. Of 1, 098 respondents, 754 (68.3%) agreed that users should consult a pharmacist before using OTC product, whereas 238 (21.6%) disagree and 112 (10.1%) had no comment on consulting the pharmacist. Of the 347 respondents who either had no comment or disagreed to consulting pharmacists, their most common reasons were unsure about the role of pharmacists, not seeing the need to consult a pharmacist, and low level of acceptance/trust to pharmacists. Multiple logistic regression showed that female gender (OR = 1.62; 95%CI 1.18-2.17; p < 0.003) and aged 18-29 years (OR = 1.99; 95%CI 1.08-3.64; p = 0.026) were two demographic factors associated with positive attitude towards pharmacist consultation on OTC products.

### Behaviour and attitude on self-care of chronic illnesses

Majority of the respondents (1024/1082; 94.6%) believed that patients with chronic illnesses should participate in self-management of their diseases, including life-style modification, routine monitoring of clinical parameters (e.g. blood pressure and blood glucose) and compliance to drug treatment. 19.5% (215/1102) respondents reported that they had chronic disease(s). The three most common chronic conditions were hypertension (n = 104; 49.1%), diabetes mellitus (n = 32; 15.1%) and hypercholesterolemia (n = 27; 12.7%). Among the respondents with chronic diseases, most of them always complied with medication (n = 188; 90.4%), complied with follow-up appointment (n = 187; 89.9%) and monitored own disease progress (n = 180, 84.5%) (Table 4). The mostly reported barriers against chronic disease self-care were lack of disease knowledge (n = 88; 43.1%), lack of monitoring equipment (n = 85; 41.5%) and unstable health status (n = 84; 40.6%) (Table 4). Patients under 60 years of age were less likely to participate in self-care of chronic diseases (OR = 0.30, 95%CI = 0.13-0.71; p = 0.006).

**Table 4:** Behaviours related to self-care and the potential barriers of respondents with chronic conditions

Frequency in performing behaviours related to self-care			
	**Always/** **Most of the time** **n (%)**	**Sometimes** **n (%)**	**Not very often/Never** **n (%)**

Disease progress monitoring#	180 (84.5%)	12 (5.6%)	21 (9.9%)
Medication compliance	188 (90.4%)	8 (3.8%)	12 (5.8%)
Clinic follow-up compliance	187 (89.9%)	7 (3.4%)	14 (6.7%)
Lifestyle modification	158 (74.9%)	28 (13.3%)	25 (11.8%)
Obtain emotional support	84 (44.7%)	27 (14.4%)	77 (41.0%)
Obtain caregiver support	124 (63.9%)	23 (11.9%)	47 (24.2%)

**Potential barriers inhibiting self-care of chronic conditions**			

	**n (%)**		
Lack of disease knowledge (n = 204)	88 (43.1%)		
Lack of monitoring equipment (n = 205)	85 (41.5%)		
Unstable health status (n = 207)	84 (40.6%)		
Lack of family/friend's support (n = 212)	70 (33.0%)		
Lack of motivation (n = 203)	62 (30.5%)		

**^#^**Measurement of blood pressure or blood glucose

### Role of pharmacist in patient self-care of chronic illnesses and self-medication

Less than half of the (497/1102; 45.1%) respondents agreed that pharmacists could play a leading role in patient self-care of chronic diseases, whereas 492 (44.6%) disagreed and 113 (10.3%) were neutral. The most common reasons for agreeing was that pharmacists facilitate medical service triage when needed (n = 206; 41.9%) and are able to monitor disease condition (n = 170, 34.6%). Those who did not agree believed that pharmacists should not take the leading role (n = 325; 67.1%) and were not familiar with the role of pharmacists (n = 80, 16.6%). Respondents in the age group of 18-29 years were more likely to support pharmacist-led patient self-care, showed by multiple logistic regression (OR = 1.6, 95%CI = 1.06-2.42; p = 0.027).

## Discussion

It is inferred from our study that approximately 60% of population in the Hong Kong community had adequate knowledge on using OTC products, primarily on western OTC products (> 60%). However, those who were at lower education level and elderly were more likely to have inadequate knowledge on using OTC products. Almost one-third (32.6%) of the respondents had purchased OTC products over the past 3 months and the majority (89.8%) claimed to follow product package instructions, which were quite consistent with the findings of the study of Wazaify and her team [18]. Our study also showed that Chinese patients with chronic diseases tended to spend more money on western and Chinese OTC products than those without chronic diseases. A study in Australia also found that 80% of their patients with a chronic condition used OTC products and many of them were not using the right dose [19]. It is therefore anticipated that patients with chronic diseases would be prone to higher risk of drug interactions between prescription drugs for chronic diseases and OTC products [20]. Based on previous studies and our research findings, elderly who received lower education and with a chronic condition are at high risk of improper use of OTC products and they are the group which needs pharmacist counselling most.

The majority (94.6%) of the population supported the practice of self-care for chronic diseases, and over 80% of those with chronic diseases claimed to perform self-care tasks regularly. Community pharmacist-provided self-care programmes had demonstrated positive impact on chronic diseases. Doucette et al. conducted a randomized controlled trial in patients with diabetes to evaluate the effect of community pharmacist-provided extended diabetes care service on patients' self-care activities [21]. Patients in the intervention group increased the number of days per week significantly (1.25 versus 0.73 days/week) engaging in diabetes diet and self-care activities. Barbanel et al. also demonstrated that patients with asthma in a self-care programme delivered by a community pharmacist had significantly better improvement of symptom scores (adjusted difference for baseline scores = 7.0 (95% CI = 4.4-9.5)) [22]. Self-care programmes provided by community pharmacists had established ground works in various chronic illnesses through health services research in western populations, yet such evidence is limited for populations in Hong Kong, China as well as other Asia regions. Though there is evidence to support the effectiveness of pharmacist-led self-care programme, only less than half (45.5%) of the population supported the pharmacist-led approach according to our study.

The evolution of the Hong Kong healthcare system and the health policy might explain why Hong Kong people have a low acceptance rate on pharmacist-led self-care management. In Hong Kong, patients receive health services from either private or public sectors seldom have the opportunity to consult community pharmacists as patients usually receive prescribed medications from private doctors directly or from government clinic pharmacies. Community pharmacists would only have the chance to provide consultation when patients visited them to buy drugs over the counter. Patients, therefore, are not familiar with the role of pharmacists besides dispensing drugs and not very supportive of pharmacist-led self-care management.

A study on the perspectives of physicians, pharmacists, traditional Chinese medicine practitioners and dispensers on patient self-care and roles of pharmacists indicated the importance of patients to self-care of their chronic conditions and they also supported pharmacists to be involved in patient self-care and take a major role in managing patients' medication issues. To provide successful continuity of care after patients return to the community, connectivity among patients, health professions and health services within the system is vital [23]. With the support of medicine professionals [24] and approximately 45% of people agreed with the pharmacist-led approach in this study, there are a few strategies that can be considered to enhance the familiarity level of patients and people in the community with pharmacists.

Community pharmacists can undertake a more active role in health promotion campaign such as drug safety in order to increase their publicity. Besides, partnership programmes can be developed between doctors and pharmacists in the community so that patients can consult pharmacists when they are not able to make their appointments with doctors. In addition, it is also necessary for the government to enhance the involvement of pharmacists in primary care and promote the roles of pharmacists through patient education, so that people can have more opportunities to communicate and contact with pharmacists.

### Limitations

The present study was limited by the relatively small number (total 37) of questions in the questionnaire. Our description of self-care activities in the survey might be inadequate. A case scenario would have provided more detailed requirements for self-care of chronic diseases and better described the role of pharmacists. The respondents therefore might have underestimated the complexity of self-care and the role of pharmacist for each common chronic disease, including hypertension, hyperlipidaemia and diabetes mellitus. In addition, those who responded to the survey could be more interested and knowledgeable about patient self-care and role of pharmacists. The views of people who refused to participate could have been neglected in this study.

## Conclusions

Over 60% of the present cohort showed adequate knowledge on using OTC products and patients with chronic diseases tended to spend more on OTC products. The majority of respondents supported self-care for chronic diseases. However less that half supported pharmacist-led self- care programmes despite the fact that elderly people and those with lower education level and a chronic condition were at high risk of encountering problems with OTC products. To overcome these limitations, self-care programmes provided by pharmacists should be gradually developed with the support of the Hong Kong SAR Government.

## Competing interests

The authors declare that they have no competing interests.

## Authors' contributions

JHY, FYW and FWC drafted the manuscripts. FYW performed data analysis. All authors were involved in the design of the study and approved the final manuscript.

## Pre-publication history

The pre-publication history for this paper can be accessed here:

http://www.biomedcentral.com/1472-6904/11/19/prepub
